# The state of international collaboration for health systems research: what do publications tell?

**DOI:** 10.1186/1478-4505-4-7

**Published:** 2006-08-23

**Authors:** Miguel A González Block

**Affiliations:** 1 Executive Director, Center for Health Systems Research, National Institute of Public Health, Av. Universidad 655 Col. Santa Maria Ahuacatitlán, Cuernavaca, 62508 Morelos, Mexico

## Abstract

**Aim:**

International collaboration for health system development has been identified as a critical input to meet pressing global health needs. North-South collaboration has the potential to benefit both parties, while South-South collaboration offers promise to strengthen capacity rapidly and efficiently across developing countries. There is an emerging trend to analyze the fruits of such collaboration. This paper builds on this trend by applying an innovative concept-based bibliometric method to identify the international scope of collaboration within the field of health policy and systems research. Two key questions are addressed: to what extent are papers comparing developing countries as against reporting on single country studies? To what extent are papers in either case being produced by researchers within their respective countries or through North-South or South-South collaboration?

**Methods:**

A total of 8,751 papers published in Medline between 1999 and 2003 with data on health systems and policies in developing countries were identified and content-analyzed using an innovative concept-based search technology. A sample of 13% of papers was used to identify the corresponding institution and countries covered. The sampled data was then analyzed by income group.

**Results:**

Papers with an international, cross-country focus account for only 10% of the total. Just over a third of all papers are led by upper middle income country authors, closely followed by authors from high income countries. Just under half of all papers target low income countries. Cross-country papers are led mostly by institutions in high income countries, with 74% of the total. Only seven countries concentrate 60% of the papers led by developing country institutions. Institutions in the United States and the United Kingdom concentrate between them as many as 68% of the papers led by high income countries. Only 11% of all single-country papers and 21% of multi-country studies are the product of South-South collaboration. Health Financing is the topic with the greatest international scope, with 26% of all papers in the topic. Topics such as Costing and Cost Effectiveness, Finance, Sector Analysis and Insurance, regardless of their national or international scope, are led in 38% to 54% of cases by high income authors.

**Conclusion:**

While there is modest health systems research capacity in many developing countries for single country studies, capacity is severely limited for multi-country studies. While North-South collaboration is important, the number of international studies is still very limited to produce the kind of knowledge required to learn from experiences across countries. The fact that lead institutions as well as study countries are concentrated in a handful of mostly middle income countries attests to great disparities in research capacity. However, disparities in research capacity and interest are also evident in the North. It is urgent to build cross-country research capacity including appropriate forms of South-South and North-South collaboration.

## Background

Collaboration for health research between developed (North) and developing (South) countries holds the potential of generating knowledge and strengthening the capacity of all parties concerned. Most North-South collaboration has been driven by developed countries and purports to benefit partners in the South [1]. However, collaborative health services research conducted in developing countries has led to significant practical and philosophical influences for health systems in the developed world [2,3]. Cross-country analysis including North and South countries has been promoted as a means of benchmarking the performance of health systems internationally, identifying limitations in specific functions and spurring improvements [4]. Comparative health systems research can also be a tool for shared learning from health sector reforms [5]. International research is a means to learn across countries and to transfer and scale-up successful interventions. The ethical dilemmas in North-South collaboration have been explored [6,7] and the best ways of approaching such collaboration have also been assessed.

International health systems research is today more necessary than ever. Global funding agencies and initiatives for disease control such as the Global Fund for AIDS, TB and Malaria and the Presidential Initiative PEPFAR are exerting increasing influence on health system strengthening and important questions are being asked as to their impact at country level [8]. Comparative and international health systems research in developing countries can be an important tool to steer such global efforts. Strategic health systems research that aims both to solve problems and to generate knowledge of international significance can benefit from research designs involving multiple countries. Importantly, strategic research capacity should exist within developing countries to ensure that high priority problems are addressed and that recommendations are taken up in a timely manner [8,9].

Yet, with few exceptions [10], no research has been undertaken to identify the situation of international, comparative health systems research. Two key questions are addressed: to what extent is the literature on health systems focusing on multiple country experiences as against reporting on single country studies? To what extent are papers in either case being produced by researchers within their respective countries or through North-South or South-South collaboration? These questions are addressed through analyzing the inter-relation of three dimensions. The first is the geographical scope of health systems papers by looking at whether they report on single or multiple countries. Multiple-country papers are in themselves evidence of international collaboration. The second dimension concerns the leadership of the studies, whether it is provided by foreign author to the study country, or in the case of multiple country papers, by an author foreign to all countries. The third dimension is the level of development of the countries studied as measured by per capita income. Through this dimension North-South or South-South collaboration can be identified. In the case of South-South collaboration, looking at the level of development allows to assess the collaboration of upper middle income, lower middle income or low income country institutions.

## Bibliometric methods

**Figure 1:**
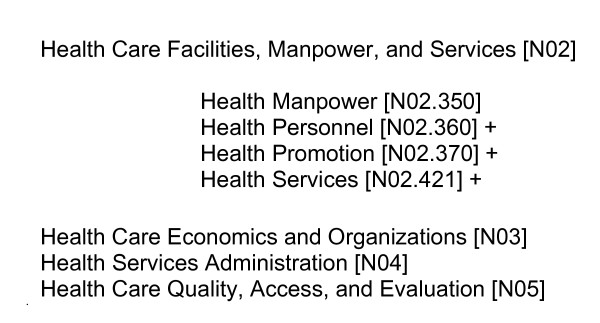
Medline MeSH Terms Used for the Primary Selection.

The study was undertaken using as the starting point Medline, a database of scientific publications in the health and medical fields published since 1991 through PubMed by the National Library of Medicine. Medline was accessed in three steps. The fist involved an advanced search in PubMed for journal articles (excluding letters, editorials and other kinds of citations) indexed by the National Library of Medicine experts under the major subject headings (MeSH) shown in figure 1. Citations were retrieved yearly from 1991 up to 2003 and for three income regions: Upper Middle Income (UMI), Lower Middle Income (LMI) and Low Income (LI). Income regions were identified by using the annual per capita income classification of the World Bank whereby LI regions have an annual per capita income of US$ 755 or less, LMIs between $756 and $2,995, and UMIs between $2,996 and $9,265.

In the second step citations where HPSR appeared as a secondary and minor topic were discarded and relevant papers were classified according to specific health systems research topics. For this step a modified version of the Evidence Base of the Alliance for Health Policy and Systems Research was used . The search engine powered by Collexis classified papers through analyzing the concepts in the abstract to produce an individualized fingerprint. Fingerprints consist of a mathematical algorithm describing the relation of concepts in the text according to frequency, proximity and concept interrelations. The set of concepts for the analysis is that of the thesaurus of the National Library of Medicine (MeSH).

**Figure 2:**
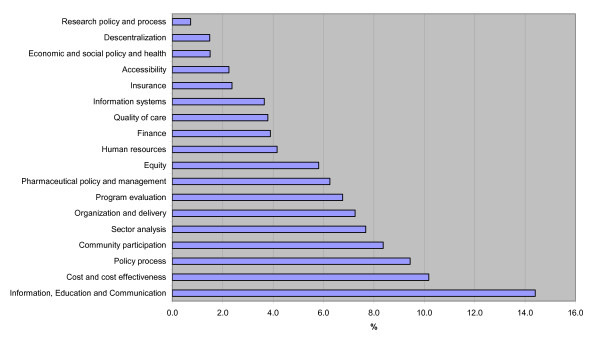
Health systems papers for developing countries, by topic.

The fingerprints for each paper were matched against the topic fingerprints developed for the field of health systems research, as follows:

1. A total of 19 health systems research topics were identified by analyzing 321 research projects undertaken by developing country researchers between 1999 and 2001 and reported to the Alliance-HPSR (Figure 2). These projects were being undertaken by 108 institutions in 39 countries and provided a good picture of developing country interests [8]. Topics were identified by focusing on the main health system functions that were the subject of inquiry [11]. Health system functions were identified at different scales of systems and by allowing cross-cutting themes. For example, a major topic "Finance" was identified, but also a minor topic "Insurance" that is conceptually a part of finance. The main criterion used to consider topics separately was that they should contain at least 2% of the total projects and that the sub-function in question was the main subject of research. Cross cutting themes such as "Community participation" included other functions or sub-functions, such as community finance and information systems, all subsumed under a different yet more prominent function within the research project.

2. A conceptual fingerprint was created for each of the 19 topics by submitting the glossary terms to the Collexis engine.

3. The topic fingerprints were then used to discard non-relevant citations and to classify the remainder. At this stage, topic fingerprints were refined through an iterative process by adding concepts found in fingerprints of highly relevant articles but absent in the topic fingerprint. This ensured that topic fingerprints captured in the end the highest number of articles with the highest relevance scores.

4. Citations were ranked according to relevance to the topic in a scale of 1% to 100%. Relevant articles were deemed to be those above 10%, as suggested by inspection of a sample of abstracts.

Table 1 shows the citations obtained at different steps in the analysis of Medline. Citations containing at least one MeSH term in the health systems research field total 87,300 for developing countries in the period 1991–2003) Of these, 9,066 or 10.4% were deemed to be relevant using the Alliance/Collexis search engine and could be classified with a fair degree of certainty within the various health systems research topics. Relevance was assessed statistically by the search engine as above 10%. The quality of selection was assessed through content analysis a selection of abstracts within each topic. Out of all papers selected, a total of 3.5% were classified in two or more topics. Once these repetitions were eliminated, a total of 8,751 papers were counted as published within health policy and systems between 1991 and 2003.

**Table 1:** Citations and hits obtained with different bibliometric procedures, Medline, 1991–2003.

	RELEVANCE*	**COUNTRY FOR WHICH RESEARCH WAS UNDERTAKEN, BY INCOME REGION**			
		
		**Low (LI)**	**Lower Medium (LMI)**	**Upper Medium (UMI)**	**TOTAL Developing Countries**
**Pre-selected citations (MeSH PubMed)**	0–100%	36,794	24,629	25,894	87,317
Topic specific, relevant citations **	11%–100%	8,039	2,467	5,729	16,235
**Unique relevant citations**	11%–100%	7,762	2,424	5,566	15,671

*As classified by the Collexis search engine. Relevance above 11% is considered acceptable for the purposes of this study.

** Topic specific hits could overlap across topics. The following row eliminates such duplications.

The concept of "Lead author" was operationalized by assuming that the author with greatest influence in the publication corresponded with the named person under the corresponding institution in the AD field in Medline. The countries studied in the paper were identified through Medline's MH field. The geographic scope of papers (single vs. multiple countries) and the income group of the countries studied and of the corresponding author were identified through quantifying the number of countries studied in each paper and through classifying them by income group. These variables were observed for a sample of papers within each of the 19 topics to enable inferences at this level. Given the exploratory nature of the study, a convenience sample of 10% of total papers was undertaken. In order to analyze proportions at the level of each topic oversampling at this level was allowed to obtain at least 40 citations per topic. This oversampling led to 242 additional papers, accounting for 24% of the total. In all, 13% of citations were sampled (Table 2). No significant changes were observed due to oversampling in the main variables at the aggregate level.

**Table 2:** Health policy and systems research papers by topic, and percentage included in the sample, Medline 1999–2003.

**TOPIC**	**In Universe**	**Sampled**	
		
		**%**	**No.**
	
Accessibility	365	11	41
Community participation	1,359	6	76
Cost and cost effectiveness	1,653	6	92
Decentralization	242	22	54
Economic and social policy and health	244	22	54
Equity	944	6	53
Finance	632	11	71
Human resources	675	11	75
Information systems	593	11	66
Information, Education and Communication	2,339	6	131
Insurance	385	11	43
Organization and delivery	1,178	6	66
Pharmaceutical policy and management	1,015	6	57
Policy process	1,533	6	86
Program evaluation	1,098	6	61
Quality of care	616	11	69
Research Policy and process	118	34	40
Sector analysis	1,246	6	70
**TOTAL**	**16,235**	7	**1,203**

## Study limitations

Only 69% of papers included the corresponding author's institutional data in the AD field, limiting the sample for this variable from 1,203 to 828 cases or 9% of the universe. Missing AD field data was more frequent for publications reporting on data for low income countries, represented in 46% of the total sample and in 39% of the citations with AD field data available. This drop is of consideration as the proportion of publications for low income countries in the universe amounts to 50%. This sampling and missing data bias implies that comparisons across income groups should be interpreted with caution.

While Medline is a very extensive database, it has well known limitations. Articles written in English account for 92% of the total while those in French and German for 2% each, Spanish for 1% and other languages for the remaining 3%. Furthermore, many journals published in developing countries and mostly of national circulation are not included. Medline also leaves out research published as internal reports or in the "grey" literature. However, the analysis of Medline is of value in itself as it reflects the knowledge that is widely available for shared learning internationally, whatever its limitations. Future studies of this type should analyze publications at the country level to include the full range of literature that can influence research as well as policy and systems development.

The corresponding author in the AD field is assumed to be the lead author. However, it may well be the case that several authors play a lead role, a fact that will not be reflected in the corresponding author. Furthermore, no consideration is taken here of the nationality of authors as this information is not available in Medline. As a result, no consideration is made of the participation of multiple authors that could be representing the different countries under study. The search was conducted to detect only papers with named developing countries in the MH field. Papers based on multiple country data not classified under a particular country will have been omitted.

## Findings

Out of a total of 828 sampled papers analyzed, 744, (90%) make reference to single developing countries and 84 (10%) make reference to multiple countries. Papers led by authors from UMI and HI countries are most frequent, with 34% and 31% of the total, respectively (bottom row in Table 3). Lead authors account for 20% and 12% of total papers in the case of LI and LMI countries, respectively. International agencies author only 3% of papers. Considering authors from HI countries and international agencies together, they account for just over one third of total papers. Looking now at the distribution of papers by the income region of the country or countries studied (last column in Table 3), those for UMI countries account for the majority, with 42%, followed by those studying LI countries, with 38% and those for LM countries with 18%. Papers including results from countries in different income regions account for 2% of the total. The disparity between papers for LI countries and the proportion of corresponding authors from this same region is quite noticeable, with 38% of papers in the literature but only 20% of corresponding authors.

**Table 3:** Coverage of papers in health policy and systems according to the income region of the corresponding author's institution.

**Medline for Developing Countries, 1991–2003**												
												
**Lead Research Institution by Region**												
**Country(ies) Studied by Region**	**HI**		**Int**		**LI**		**LMI**		**UMI**		**TOTAL**	
	**No.**	**%**	**No.**	**%**	**No.**	**%**	**No.**	**%**	**No.**	**%**	**No.**	**%**
**LI**	127	49	18	69	164	99			7	2	**316**	**38**
**LM**	50	19	3	12			93	96	1	0	**146**	**18**
**UMI**	74	29	5	19	1	1			267	95	**348**	**42**
**Mixed**	6	2			1	1	4	4	7	2	**18**	**2**
**TOTAL**	**257**	**100**	**26**	**100**	**166**	**100**	**97**	**100**	**282**	**100**	**828**	**100**
		**31**		**3**		**20**		**12**		**34**		**100**

Looking now in more detail at Table 3, in the case of papers led by authors from HI countries, just under half or 49%, targeted LI countries while 29% targeted UMI countries and the remaining 19% LMI countries. International agencies focus more on LI countries, with 69% of papers led by authors from these agencies. Papers led by authors from all developing country regions focus almost exclusively on their own region (and country, as will be seen below), with between 95% and 99% of cases.

How is the authorship of single vs. multiple country papers distributed across regions? In the case of single country papers, UMI lead authors account for the most, with 37% of the total, followed about equally by HI and LI authors and then by LMI authors (bottom row of Table 4). In the case of multiple country papers (bottom row of Table 5), HI lead authors account for 74% of total, authors from international agencies for 5%, while developing country authors account for the remaining 21%, with UMI authors accounting for half of these.

**Table 4:** Country coverage of single-country papers in health systems according to the nationality and income region of the corresponding author's institution.

**Medline for Developing Countries, 1991–2003**														
														
**Country of research institution, by region**														
**Lead author's institution**	**Country studied, by region**	**HI**		**Int.**		**LI**		**LMI**		**UMI**		**TOTAL**		
		**No.**	**%**	**No.**	**%**	**No.**	**%**	**No.**	**%**	**No.**	**%**	**No.**	**%**	
**Is from country studied**	**LI**	-	-	-	-	153	94					**153**	21	
	**LM**	-	-	-	-			90	98			**89**	12	**66**
	**UMI**	-	-	-	-					247	90	**248**	33	
**Is foreign to country studied**	**LI**	99	51	14	64	9	6			6	2	**128**	17	
	**LM**	43	22	3	14			2	2	1	0	**49**	7	**34**
	**UMI**	53	27	5	23					19	7	**77**	10	
**Subtotal**		195	100	22	100	162	100	92	100	273	100	744	100	100
			**26**		**3**		**22**		**12**		**37**		**100**	

**Table 5:** Countries covered by multiple-country health policy and systems papers, according to the nationality and income region of the lead author's institution.

**Medline for Developing Countries, 1991–2003**														
														
**Country of research institution, by region**														
														
**Lead author's institution**	**Countries studied, by region**	**HI**		**Int.**		**LI**		**LMI**		**UMI**		**Total**		
		**No.**	**%**	**No.**	**%**	**No.**	**%**	**No.**	**%**	**No.**	**%**	**No.**	**%**	
**Is from one of study countries**	**LI**	11	18	-	-	2	50					**13**	**15.5**	
	**LM**	3	5	-	-			1	20			**4**	**4.8**	
	**UMI**	17	27	-	-	1	25			1	11	**19**	**22.6**	**57**
	**Several regions**	1	2	-	-	1	25	4	80	6	67	**12**	**14.3**	
**Is foreign to all study countries**	**LI**	17	27	4	100					1	11	**22**	**26.2**	
	**LM**	4	6									**4**	**4.8**	**43**
	**UMI**	4	6									**4**	**4.8**	
	**Several regions**	5	8							1	11	**6**	**7.1**	
**Subtotal**		**62**	**100**	**4**	**100**	**4**	**100**	**5**	**100**	**9**	**100**	**84**	**100**	**100**
			**74**		**5**		**5**		**6**		**11**		**100**	

To what extent are papers the fruit of foreign collaboration or leadership? This can be answered by analyzing whether the lead author's institution is foreign to the country or countries studied. In the case of multiple country studies led by HI authors, it is relevant to ask whether their country is included or not in the study.

The frequency of papers involving foreign collaboration is 34% in the case of single country studies (last column in Table 4) and 43% in the case of multi-country studies (last column in table 7). Who is leading this collaboration? In the case of single country papers, foreign collaboration came mostly from HI countries, with 77% of the total (second to last row in table 6). International agency authors led 9% of collaborations while developing country authors led the remaining 15%. In the case of multiple country papers, 83% of foreign collaboration came from HI authors, 11% came from international agency authors and only 6% from UMI authors. No multi-country papers led by LI or LMI were reported. Analyzing further the collaboration by HI authors in multi-country papers, it can be seen that in 52% of cases the author's institution was included. In these cases, an important reason to engage in multi-country work may have been to derive lessons for the HI country in question. In the remainder 48% of cases HI support may have been more directly related to support developing countries.

**Table 6:** Number of countries covered by papers.

	**Country of lead author, by region**											
**No. of Countries**	**HI**		**Int.**		**LI**		**LMI**		**UMI**		**TOTAL**	
	
	**No.**	**%**	**No.**	**%**	**No.**	**%**	**No.**	**%**	**No.**	**%**	**No.**	**%**

**One**	195	76	22	85	162	98	93	96	273	97	**745**	**90**
**Two**	41	16	3	12	3	2	4	4	7	2	**58**	**7**
**Three to five**	21	8	1	4	1	1			2	1	**25**	**3**

**TOTAL**	**257**	**100**	**26**	**100**	**166**	**100**	**97**	**100**	**282**	**100**	**828**	**100**

**Table 7:** Health systems papers by country of corresponding author institution. Developing Countries, Medline 1991–2003*.

**Low Income**			**Lower Middle Income**			**Upper Middle Income**			**High Income**		
	No.	%		No.	%		No.	%		No.	%
India	52	31	China	46	48	Brazil	93	33	USA	121	47
Nigeria	24	14	Thailand	19	20	South Africa	62	22	United Kingdom	54	21
Kenya	16	9.6	Colombia	6	6.3	Mexico	31	11	Sweden	13	5.1
Ethiopia	10	6.0	Papua New Guinea	5	5.2	Korea	17	6.0	Australia	12	4.7
Pakistan	9	5.4	Philippines	4	4.2	Turkey	15	5.3	Canada	8	3.1
Vietnam	9	5.4	Tunisia	3	3.1	Mexico	12	4.3	France	8	3.1
Bangladesh	8	4.8	Cuba	2	2.1	Chile	9	3.2	Germany	7	2.7
Uganda	8	4.8	Egypt	2	2.1	Saudi Arabia	9	3.2	Italy	6	2.3
Tanzania	7	4.2	Jamaica	2	2.1	Argentina	7	2.5	Netherlands	5	1.9
Zimbabwe	5	3.0	Kazakhstan	2	2.1	Malaysia	7	2.5	Switzerland	5	1.9
Ghana	2	1.2	Peru	2	2.1	Puerto Rico	7	2.5	Japan	4	1.6
Indonesia	2	1.2	Iran	1	1.0	Oman	3	1.1	Belgium	3	1.2
Burkina Faso	1	0.6	Jordan	1	1.0	Lebanon	2	0.7	Portugal	3	1.2
Guinea-Bissau	1	0.6	Malaysia	1	1.0	Bahrain	1	0.4	Denmark	2	0.8
Honduras	1	0.6				Botswana	1	0.4	Norway	2	0.8
Lesotho	1	0.6				Croatia	1	0.4	Finland	1	0.4
Madagascar	1	0.6				Israel	1	0.4	Ireland	1	0.4
Mali	1	0.6				Panama	1	0.4	New Zealand	1	0.4
Mozambique	1	0.6				Trinidad and Tobago	1	0.4	Spain	1	0.4
Myanmar	1	0.6				Uruguay	1	0.4			
Nicaragua	1	0.6				Venezuela	1	0.4			
Niger	1	0.6									
North	1	0.6									
Senegal	1	0.6									
Uzbekistan	1	0.6									
Zambia	1	0.6									

TOTAL	166	100		96	100		282	100		257	100

Total Countries	26	16		14			21			19	

*28 International agencies are excluded in this table

Regardless of the origin of expertise, a large proportion of publications demonstrate some form of international collaboration or interest. This is evidently the case of papers with a clear cross-country scope. However, international collaboration is also shown by single country papers with a foreign lead author, accounting for 41% of total papers. Papers led by authors from foreign institutions, whether single or multi-country, account for 35% of the total. However, international expertise for multi-country studies is heavily concentrated in HI countries and international agencies, where 79% of papers are lead authored.

What is the range of countries studied in multi-country papers? (Table 6). Out of all papers, as already stated, 90% are single-country studies. Of the remaining 10%, they are mostly two-country studies, with 7% of the total, while the remaining 3% include more than two. The one study sampled with most countries included 5. Most multi-country papers were lead by authors from HI countries and from international agencies.

What is the country distribution of papers? A total of 80 countries have institutions in the lead author position, of which 59 are in developing countries and 21 in high income countries (Table 7). There is a heavy concentration in a handful of countries. Only seven countries: India, Nigeria, China, Thailand, South Africa, Brazil and Mexico, concentrate 60% of the papers led by developing country authors. The United States and the United Kingdom concentrate between them as many as 68% of the papers led by high income countries and account for 21% of all papers (including those by international agencies, not shown in Table 5). This suggests there is a marked concentration of expertise in health systems research and in international health in a few countries and institutions.

The countries actually studied in the sampled papers total 106, of which 94 are developing countries and 12 are high income countries included as part of multi-country papers (Table 8). However, the concentration of countries studied is still very high, with the same seven developing countries mentioned above accounting for 47% of total papers and 19 developing countries, or 20% of the total, accounting for 70% of papers.

**Table 8:** Countries studied in papers including at least one developing country. Medline 1999–2003.

**Low Income**			**Lower Middle Income**			**Upper Middle Income**			**High Income**			**Total**
**Country**	**No.**	**%**	**Country**	**No.**	**%**	**Country**	**No.**	**%**	**Country**	**No.**	**%**	
India	148	23.1	China	81	37.7	Brazil	134	27.3	USA	52	52.5	
Nigeria	47	7.3	Thailand	32	14.9	South Africa	110	22.4	Great Britain	15	15.2	
Tanzania	43	6.7	Philippines	13	6.0	Mexico	84	17.1	Canada	7	7.1	
Vietnam	40	6.2	Colombia	11	5.1	Korea	28	5.7	Japan	6	6.1	
Kenya	38	5.9	Egypt	11	5.1	Turkey	28	5.7	Netherlands	6	6.1	
Bangladesh	36	5.6	Guatemala	9	4.2	Chile	25	5.1	England	2	2.0	
Uganda	26	4.0	Papua New Guinea	9	4.2	Puerto Rico	19	3.9	France	2	2.0	
Pakistan	23	3.6	Morocco	7	3.3	Argentina	13	2.7	Germany	2	2.0	
Zimbabwe	23	3.6	Peru	7	3.3	Malaysia	13	2.7	Italy	2	2.0	
Ghana	19	3.0	Tunisia	7	3.3	Saudi Arabia	10	2.0	New Zealand	2	2.0	
Zambia	18	2.8	Jordan	5	2.3	Botswana	4	0.8	Switzerland	2	2.0	
Indonesia	17	2.6	Dominican Republic	4	1.9	Lebanon	4	0.8	Sweden	1	1.0	
Nepal	17	2.6	Cuba	3	1.4	Venezuela	4	0.8				
Burkina Faso	14	2.2	Jamaica	3	1.4	Panama	3	0.6				
Ethiopia	14	2.2	Kazakhstan	3	1.4	Georgia	2	0.4				
Rwanda	10	1.6	El Salvador	2	0.9	Oman	2	0.4				
Mali	8	1.2	Iran	2	0.9	Bahrain	1	0.2				
Nicaragua	8	1.2	Belize	1	0.5	Gabon	1	0.2				
Senegal	8	1.2	Brazil	1	0.5	Iraq	1	0.2				
Benin	7	1.1	Cambodia	1	0.5	Morocco	1	0.2				
Mozambique	7	1.1	Costa Rica	1	0.5	Trinidad and Tobago	1	0.2				
Cameroon	6	0.9	Ecuador	1	0.5	Uruguay	1	0.2				
Cote d'Ivoire	6	0.9	Namibia	1	0.5	Uzbekistan	1	0.2				
Haiti	6	0.9										
Niger	6	0.9										
Malawi	5	0.8										
Cambodia	4	0.6										
Burundi	3	0.5										
Chad	3	0.5										
Gambia	3	0.5										
Myanmar	3	0.5										
Sri Lanka	3	0.5										
Afghanistan	2	0.3										
Congo	2	0.3										
Honduras	2	0.3										
Lesotho	2	0.3										
Madagascar	2	0.3										
Sudan	2	0.3										
Swaziland	2	0.3										
Bhutan	1	0.2										
Central African Republic	1	0.2										
Democratic Republic of the Congo	1	0.2										
Guinea-Bissau	1	0.2										
Kyrgyzstan	1	0.2										
Laos	1	0.2										
Liberia	1	0.2										
Somalia	1	0.2										
Togo	1	0.2										

**Total**	**642**	**100**		**215**	**100**		**490**	**100**		**99**	**100**	**1446**

**Total countries in sample**	**48**			**23**			**23**			**12**		**106**

To what extent is leadership related to the specific topic of the papers? It is worth noting first, however, that topics with the greatest number of papers are those for Information, Education and Communication, Community Participation, Costing and Cost Effectiveness and Policy Process, with between 9.4% and 14.4% of all topics (figure 2). Topics with lowest frequency are Research Policy and Process, Decentralization and Economic and Social Policy and Health, with between 0.7% and 1.5% of total papers. The frequency of topics is analyzed in greater detail elsewhere [8].

Of all papers, Health Financing has the greatest international scope, with 26% of all papers within the topic (Table 9). This figure then drops from 14% to 18% for papers on Information, Education and Communication (IEC), Equity, Human Resources and Research Policy and Process. Topics with very few cross-country studies, at only 3% to 6%, are Community Participation, Costing and Cost Effectiveness and Program Evaluation. There is a negative association between the frequency of topics in the total sample and the frequency of cross-country papers within each topic (corr = -0.27).

**Table 9:** Country coverage of health systems papers by topic. Medline 1991–2003*.

**TOPIC**	**Coverage of Study**					
	
	**Single country**		**International**		**TOTAL universe**	
	
	**No**	**%**	**No**	**%**	**No**	**%**
Financing	262	74	91	26	353	100
Research policy and process	54	82	12	18	66	100
Human resources	313	83	64	17	377	100
Equity	453	86	74	14	527	100
Information, Education and Communication	1,125	86	181	14	1,306	100
Policy process	741	87	115	13	856	100
Insurance	187	87	28	13	215	100
Economic and social policy and health	121	89	15	11	136	100
Sector analysis	625	90	71	10	696	100
Accessibility	185	90	19	10	204	100
Information systems	300	91	31	9	331	100
Descentralization	123	91	13	9	135	100
Quality of care	313	91	31	9	344	100
Pharmaceutical policy and management	516	91	51	9	567	100
Organization and delivery	607	92	51	8	658	100
Program evaluation	573	94	40	6	613	100
Cost and cost effectiveness	884	96	39	4	923	100
Community participation	740	97	19	3	759	100

**TOTAL**	**8,121**		**945**		**9,066**	

Topics show important differences with respect to the income region of the lead author (Table 10). Topics such as Costing and Cost Effectiveness, Finance, Sector Analysis and Insurance are led in 38% to 54% of cases by high income authors. At the other end, topics such as Policy Process, Programme Evaluation and Quality of Care are only led by such authors in 21% to 22% of cases. This data suggest a concentration of expertise in the North for certain subjects, particularly health economics. There is a positive association between the frequency with which topics are treated internationally and the extent of leadership by authors from HI (corr = 0.29).

**Table 10:** Health systems papers by income region or international agency origin of corresponding author and by topic medline 1991–2003*.

**TOPIC**	**Corresponding author's income region/Int.agency**						**TOTAL**	
			
	**High Income**		**Int. Agency**		**Developing country**			
			
	**No**	**%**	**No**	**%**	**No**	**%**	**No**	**%**
Accessibility	63	31	10	5	131	64	204	100
Community participation	190	25	27	4	542	71	759	100
Cost and cost effectiveness	346	38	69	8	508	55	923	100
Descentralization	42	31	3	2	90	67	135	100
Economic and social policy and health	38	28	-	-	98	72	136	100
Equity	169	32	21	4	337	64	527	100
Finance	158	45	9	3	186	53	353	100
Human resources	105	28	7	2	265	70	377	100
Information systems	81	25	12	4	238	72	331	100
Information, Education and Communication	435	33	17	1	853	65	1,306	100
Insurance	115	54	5	2	94	44	215	100
Organization and delivery	212	32	24	4	423	64	658	100
Pharmaceutical policy and management	145	25	11	2	411	73	567	100
Policy process	180	21	68	8	608	71	856	100
Program evaluation	134	22	22	4	457	75	613	100
Quality of care	76	22	-	-	268	78	344	100
Research Policy and process	17	25	-	-	50	75	66	100
Sector analysis	316	45	47	7	332	48	696	100

**TOTAL**	**2,821**		**353**		**5,892**		**9,066**	

* Projected on the basis of a sample of 13% of all papers that were content analyzed.

Looking now at the frequency of international papers by topic, the greatest number of them belongs to IEC, with 181 or 14 per year. This is followed by Policy Process, with 115 or 13 per year. The topic of Financing, in spite of having the greatest percentage of international publications, accounts for 91 total papers or 9 per year. Topics such as Decentralization, Economic and Social Policy, Community Participation and Accessibility account for less than 2 international papers per year on average.

## Conclusion

The utilization of the concept-based search engine enabled the reliable analysis of a whole field of interdisciplinary research. This methodology proved more robust than the classification of papers through the Medline MeSH key-words, which tend to include papers with only marginal interest for the field. Furthermore, this methodology enabled the topic-specific analysis of the field, identifying the specific areas where international collaboration is stronger and where international papers are more frequent.

The fact that up to two thirds of the total health systems papers for developing countries are led by developing country authors indicates the existence of a minimum of research capacity in many developing countries. On the other hand, the one third of papers led by HI authors and to a lesser extent by international agencies points to the importance of North-South collaboration. This form of collaboration gives greater emphasis to LI countries, although close to one third of these papers involve UMI countries. Given marked disparities in research capacity across income regions in the developing world, a higher proportion of North-led papers would be expected to focus in LI countries. Given also the expertise in health systems within international agencies such as WHO, the fact that only 3% to 5% (for single and multiple country studies, respectively) of all publications are led by such agencies suggests a missed opportunity to disseminate in scientific journals and to make the most of available data.

South-South collaboration in health systems analysis and research is at a very low level. Only 11% of all single-country papers led by foreigners (which account for 34% of the total) are led by developing country institutions. Capacity to lead multi-country studies is also low in developing countries, with only 21% of such studies and virtually absent in the case of foreign-led studies.

Multi-country studies, with only 10% of the total and around 115 papers per year are scarce no matter who undertakes them, and may not be realizing the full potential to generate the needed knowledge for health systems development. While the figure of cross-country studies is encouraging for some topics such as Health Financing, most of these papers are two-country comparisons, perhaps not extensive enough to generate knowledge on trends and determinants that can be generalized beyond the study countries.

While minimum research capacity may exist in many developing countries, the fact that lead institutions as well as study countries are concentrated in a handful of mostly middle income countries attests to great disparities in research capacity. However, disparities are also evident in the North, where just two institutions, the United States of America and the United Kingdom, concentrate 60% of papers led by high income countries and produce one fifth of all the literature indexed in Medline on health system research. Paper leadership by high income country institutions has important differences across topics, with a marked emphasis in health economics. This finding suggests a particular lack of capacity in this area in the developing world, although it could also be the result of a marked interest in health economics on the part of institutions in high income countries.

This paper attests to the small part played by comparative health systems literature and by papers with an international focus in the context of Medline. Furthermore, the analysis demonstrates the large role played in this respect by Northern authors. However, this analysis says nothing on the actual influence of the literature on the research agendas or on policy. Further research is required to answer these questions, in the light of what is known about how research projects actually influence on the research agendas and on health policy [8].

Regional research networks have been encouraged to strengthen capacity and to promote comparative research. However, evidence shows that the limiting factor in this kind of research is funding. Indeed, current funding averaging around US$ 20,000 is too low even to ensure successful single country research [8]. It is likely that successful multi-country research projects will require funding in the order of US$500,000 per project, to involve three to four countries. This is a small amount considering the current funding levels for health system strengthening at the global level.

It is urgent to build capacity to generate knowledge based on cross-country research. North-South collaboration can be strengthened by promoting further involvement of countries such as Germany, France, Belgium and Spain in funding, supporting and leading research in developing countries. However, South-South collaboration has to be strengthened through innovative strategies. Institutions in upper middle income countries that are now concentrating most comparative research capacity can be encouraged and supported to lead research involving low income countries. This involvement can ensure not only technical capacity, but also a more appropriate research process leading to effective policy impact.
